# *Angelica sinensis* (Oliv.) Diels: Influence of Value Chain on Quality Criteria and Marker Compounds Ferulic Acid and *Z*-Ligustilide

**DOI:** 10.3390/medicines4010014

**Published:** 2017-03-14

**Authors:** Nino Giacomelli, Yang Yongping, Franz K. Huber, Anita Ankli, Caroline S. Weckerle

**Affiliations:** 1 Institute of Systematic and Evolutionary Botany, University of Zurich, Zollikerstrasse 107, 8008 Zurich, Switzerland; n_giacomelli@hotmail.de (N.G.); huber@ethnobot.ch (F.K.H.); 2 Kunming Institute of Botany, Chinese Academy of Sciences, No. 132, Lanhei Road, Kunming 650201, Yunnan, China; yangyp@mail.kib.ac.cn; 3 CAMAG Laboratory, Sonnenmattstrasse 11, 4132 Muttenz, Switzerland; anita.ankli@bluewin.ch

**Keywords:** *Angelica sinensis*, Chinese medicine, *dang gui*, ethnobotany, Gansu, medicinal plants, TCM, Yunnan

## Abstract

**Background:** *Dang gui* (Apiaceae; *Angelica sinensis* radix) is among the most often used Chinese medicinal plants. However, hardly anything is known about its value chain and its influence on the main marker compounds of the drug. The aim of this study is to investigate the value chain of *dang gui* in Gansu and Yunnan, and the analysis of the marker compounds ferulic acid and Z-ligustilide concentration in relation to quality criteria such as the production area and size of the roots. **Methods:** During six months of field research in China, semi-structured interviews with various stakeholders of the value chain were undertaken and plant material was collected. High-performance thin layer chromatography (HPTLC) was used for semi-quantitative analysis of ferulic acid and Z-ligustilide. **Results:** Small-scale household cultivation prevails and in Gansu—in contrast to Yunnan—the cultivation of *dang gui* is often the main income source of farmers. Farmers and dealers use size and odor of the root as main quality criteria. For Chinese medicine doctors, Gansu as the production area is the main criterion. Higher amounts of ferulic acid in plant material from Yunnan compared to Gansu were found. Additionally, a negative relation of root length with both ferulic acid and Z-ligustilide as well as head diameter with ferulic acid were found. **Conclusions:** HPTLC is a valid method for semi-quantitative analysis of the marker compounds of *dang gui*. However, the two main marker compounds cannot explain why size and smell of the root or production area are seen as quality criteria. This hints at the inherent difficulty to correlate quality notions of medicinal plants with specific chemical compounds. With respect to this, more attention should be paid to quality in terms of cultivation and processing techniques.

## 1. Introduction

### 1.1. Angelica sinensis Traditional Use as Medicine in China

The use of *Angelica sinensis* (Oliv.) Diels roots (Apiaceae; Radix *Angelicae sinensis*)—in Chinese called *dang gui*—can be traced back almost 2000 years to the “Divine Husbandman’s Classic of Materia Medica” (*Shen nong ben cao jing*), and is one of the most commonly used and widely cultivated Chinese medicines [1,2]. Nowadays, more than 70 formulas containing *A. sinensis* are recorded in the Chinese pharmacopoeia [3,4].

*Dang gui* can be divided into three distinct parts, namely head, body, and tail, which are reported to have different therapeutic effects. The head is mainly used to stop bleeding, the body to nourish the blood and the tail to quicken the blood [2]. The therapeutic actions that are historically attributed to *dang gui* are summarized in Table 1 and juxtaposed with recent pharmacological studies.

Of the more than 70 isolated and identified compounds from *dang gui*, ferulic acid, Z-ligustilide and other phthalides are most often associated with the pharmacological activities [5,6] and commonly used marker components for the quality assessment of *dang gui* [2,6,7]. High-performance thin layer chromatography (HPTLC) is the method of choice in the pharmacopoeia of the People’s Republic of China (2012) for the identification of *dang gui*. However, recent analytical studies focusing on the quantification of the marker components ferulic acid and Z-ligustilide mainly apply gas chromatography coupled with mass spectrometry (GC-MS) or high-performance liquid chromatography (HPLC) [2,6,8]. The quantification of these two components can also be carried out by HPTLC, but this has not yet been done for *dang gui* [9].

### 1.2. Value Chain and Research Questions

Recent studies on turmeric (*Curcuma longa* L., Zingiberaceae) have shown that the value chain of a plant product has a major influence on its quality and chemical composition [10]. No studies published in international journals are available on the value chain of *dang gui*. This study aims at filling this gap and is interested in the value chain of *dang gui* from the farmer to the shop. It uses HPTLC to assess raw and processed material and analyses the concentration of the marker components in relation to the production area, size and processing of the roots, and price of material. The following questions are answered: What do participants of the value chain know about *A. sinensis* roots? Are there differences concerning the abundance of ferulic acid and Z-ligustilide? Has processing such as slicing and sulfuring an impact on the concentration of ferulic acid and Z-ligustilide? How is the root size linked to the content of ferulic acid and Z-ligustilide?

## 2. Study Sites

### 2.1. Cultivation of Dang Gui

*Angelica sinensis* naturally grows in cool, high-altitude mountainous regions between 2500 m and 3000 m in Gansu, Hubei, Shaanxi, Sichuan and Yunnan Provinces [26]. It is cultivated in several provinces of China, namely Qinghai, Gansu, Sichuan and Yunnan (production status investigation in 1998). In Gansu, the cultivation of *dang gui* mainly takes place in the south and accounts for 90% of the total Chinese *dang gui* production [6]. Among the cultivation areas in Gansu, Min County has the largest yield, producing more than 6000 tons, which makes up 70% of the nationwide production and 80% of the exported material. *Dang gui* cultivated in Gansu is thought to be of best quality and is regarded as an authentic and superior medicinal material (*Daodi yaocai*) [27].

Production of *dang gui* in Gansu played an important role in the local economic development and still occupies a strong position in agricultural production [28].

Yunnan has the second largest production of *dang gui* [29]. Like other places, Yunnan introduced *dang gui* from Gansu in the 19th century. According to records, *dang gui* was first introduced to Lanping County during the Qing dynasty around 1815–1850 [30]. In Yunnan, the cultivation of *dang gui* is restricted to two geographically separated regions located in the northwest and in the east of the province.

Fieldwork for this investigation on *dang gui* took place in Gansu and Yunnan Provinces.

### 2.2. Gansu Province

Gansu is located at the juncture of the Qinghai-Xizang Plateau, Loess Plateau and the Mongolia-Xinjiang Plateau in the northwest of China. High mountains, river valleys, plains, deserts, grasslands and forests (10% of the area) make up the diverse landscape [31]. The continental climate is semi-arid to arid [32]. Local ethnic groups include Hui, Dongxia and Tibetans among others.

### 2.3. Yunnan Province

Yunnan is located in the southwest of China. This region features a great variety of landscapes ranging from subtropical rainforests to alpine landscapes, coupled with an extremely high biodiversity [33]. A large number of ethnic groups with distinct languages and customs inhabit this province and account for one-third of the total population [34]. These include, among others, the Yi, Bai, Hani, Dai, Lisu and Naxi.

## 3. Material and Methods

### 3.1. Fieldwork

Fieldwork took place from July to December 2014. First, general information about the *dang gui* business and value chain as well as current places of production was gathered by means of informal interviews at the market for Chinese medicine in Kunming *Xinluosiwan* wholesale market for Chinese medicine.

In total, 118 semi-structured interviews with farmers, dealers, Chinese medicine doctors and lay people (consumers) were conducted in 11 villages in southern Gansu, eastern and northwestern Yunnan, and in Kunming (Table 2). In Gansu, the two most famous villages for the cultivation of *dang gui* were visited. As there was only little information available about the exact cultivation locations in Yunnan, the itinerary heavily relied on information of informants and was perpetually adjusted. All semi-structured interviews were conducted in Chinese with the help of a Chinese translator.

The most detailed interviews were done with farmers (*n* = 22), on plant knowledge, cultivation, value chain and livelihood issues. Most of the farmers were middle-aged (M = 42 years, SD = 11 years) and male (19 men, 3 women). The vast majority of the farmers was Han Chinese (*n* = 15), and a few farmers were Bai (*n* = 5) or Lisu (*n* = 2). Interviewees were exclusively chosen by snowball sampling [35]. In most cases, the interviewed farmers were willing to dig out an *Angelica sinensis* plant, which was then vouchered.

In total, 28 interviews with dealers (16 men, 12 women), without gathering information about age, were conducted at the market for Chinese medicine in Kunming. Main questions were about quality, demand and prices of *dang gui*. The interviews with Chinese medicine doctors (*n* = 14, 12 men, 2 women) were conducted in doctor’s offices scattered all over Kunming. Main questions were about quality and specific uses of the plant. Some doctor’s offices were shared among Chinese medicine (CM) and Western medicine practitioners, while in most cases CM practitioners had their own office. Again, no data about age of the interviewees was gathered. For the interviews with dealers and CM doctors, a convenience sampling attempt was chosen. In case of the interviews with consumers, a quota sampling was done, as sex of the interviewees was taken into consideration [35]. These interviews were conducted within the botanical garden of the Kunming Institute of Botany with a total of 54 visitors (27 women, 27 men, average age = 42 years, SD = 17 years). They were questioned about their attitude towards Chinese medicine in general and their use and knowledge of *dang gui*.

Research was conducted in agreement with the Convention on Biological Diversity (CBD; [36]), including the Bonn Guidelines on Access and Benefit Sharing (ABS).

### 3.2. Plant Material

**Figure 1:**
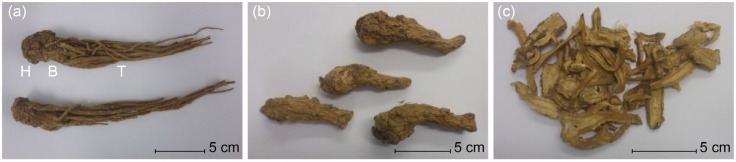
(**a**) Whole root of *Angelica sinensis* (Quangui) with head (H), body (B), and tail (T); (**b**) The combined head and body are nowadays commonly sold as head (Guitou); (**c**) Sliced *dang gui* (Guipian).

Most of the samples were purchased at local markets. We always asked the respective dealers about the place of cultivation, selling price (without bargaining), and whether the material was sulfured. Altogether, more than 200 samples were collected. These consist of whole roots (Quangui; Figure 1a; 101 samples from Gansu and 79 from Yunnan), upper part of the root (Guitou; Figure 1b; 5 samples from Gansu and 14 from Yunnan) and sliced roots (Guipian; Figure 1c; 10 samples from Gansu). Today, the combined head and body are sold as head, but in former times there was a clear distinction between the two [25]. In the analytical part of this work, head does not refer to the combination of head and body, but to the distinct uppermost part of the root.

Voucher specimens were deposited at the herbarium of the Institute of Systematic and Evolutionary Botany, University of Zürich (Z). Nomenclature follows *The Plant List* ([37]) and *Flora of China* ([38]).

### 3.3. High Performance Thin Layer Chromatography (HPTLC) Analysis

**Figure 2:**
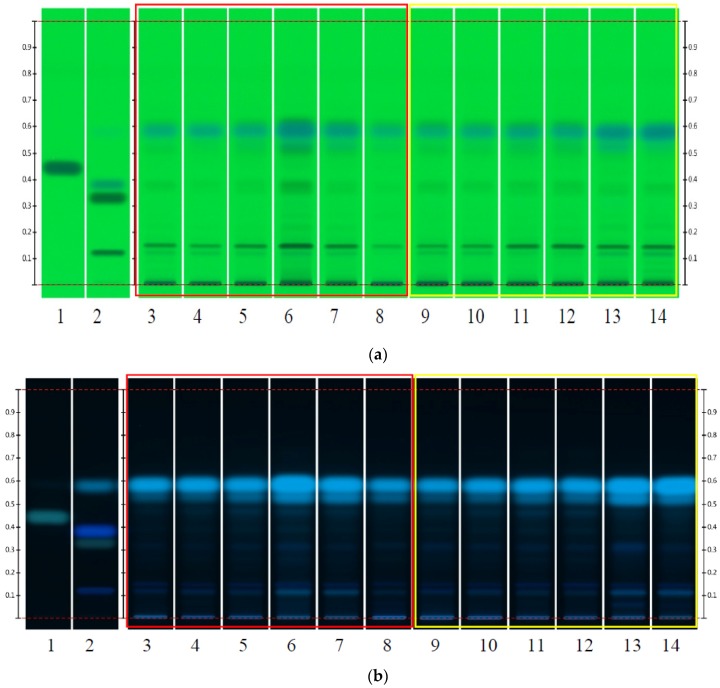

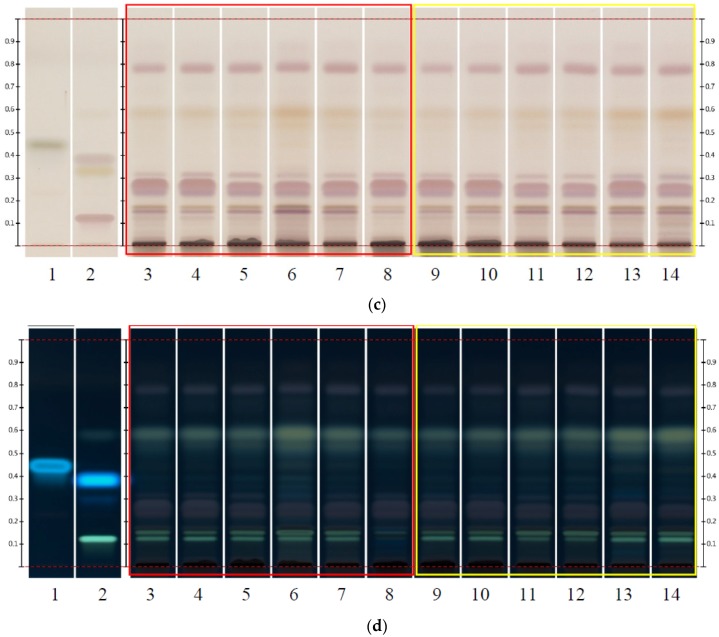
HPTLC fingerprints of *Angelica sinensis* cultivated in Gansu (tracks 3–8) and Yunnan (tracks 9–14). Isoimperatorin (R_f_ = 0.46), Imperatorin (R_f_ = 0.38), ferulic acid (R_f_ = 0.12), osthole (R_f_ = 0.33) and Z-ligustilide (R_f_ = 0.59) were used as reference substances. (**a**) Image of the plate prior to derivatization under UV 254 nm; (**b**) Image of the plate prior derivatization under UV 366 nm; (**c**) Image of the plate after derivatization under WRT; (**d**) Image of the plate after derivatization under UV 366 nm.

The HPTLC analysis was carried out in the laboratory of the headquarters of CAMAG in Muttenz, Switzerland. For all experiments, roots were first treated as a single unit and hence processed as whole root. Afterwards, samples belonging to the same batch were pooled or selected roots were cut into pieces. All samples were processed as follows: Roots were cut and ground for 2.5 min at 14,200 rpm to a moderately fine powder (sieve size 355). The unsieved remains were stored and later processed similarly to the sieved samples. Of each sample, 1 g was mixed with 4 mL of methanol and shaken for 10 min at 200 rpm. Samples were centrifuged at 14,200 rpm for 5 min and the supernatant was transferred into vials. Isoimperatorin, imperatorin, osthole, Z-ligustilide and ferulic acid (PhytoLab GmbH & Co. KG, Vestenbergsgreuth, BY, Germany) were used as reference substances. An amount of 1 mg of each isoimperatorin, imperatorin, osthole and ferulic acid were dissolved in 1 mL of methanol, and 10 µL of Z-ligustilide were dissolved in 1 mL of methanol. By means of the automatic TLC sampler (ATS4, CAMAG, Muttenz, BL, Switzerland), 4 μL of isoimperatorin, imperatorin, osthole, Z-ligustilide and 2 μL of ferulic acid were applied on the plate (HPTLC Si 60 F_254,_ MERCK, Darmstadt, HE, Germany). Of all the sample solutions, 4 μL were applied. The plates were subsequently developed in the automatic developing chamber (ADC2, CAMAG, Muttenz, BL, Switzerland), whereas the chamber was saturated, the developing distance set to 70 mm from the lower edge, and the relative humidity set to 33% (MgCl_2_). A mixture of toluene, ethyl acetate and glacial acetic acid at a ratio of 90:10:1 (*v*/*v*/*v*) was used as developing solvent. For identification of compounds, the plate was dipped into a sulfuric acid solution (10% sulfuric acid in methanol, CAMAG Chromatogram Immersion Device III; speed: 3, time: 0) and heated at 100 °C for 5 min. For the purpose of documentation, pictures of the plates were taken prior to derivatization under white RT light (R = remission, T = transmission), UV 254 nm and 366 nm and white RT after derivatization, respectively (Figure 2). To examine the differences among parts within the root, 20 roots from one batch were divided into head, body and tail according to verbal instructions given by Chinese doctors and analyzed separately.

In order to assess the repeatability of the method, three samples of powdered material were extracted three times and each extract applied three times on three different plates. The relative standard deviation was then calculated for each sample within the plate. To determine loss on drying, which was carried out following the instruction of the European Pharmacopoeia ([39]), 12 representative samples were selected. Thereafter, 1 g of each powdered sample was heated at 105 °C in an oven for 2 h. For the semi-quantitative assessment, a single-level calibration of each standard, ferulic acid and Z-ligustilide were prepared in the following concentration: 1 mg/mL of ferulic acid and 10 µL/mL for Z-ligustilide. The profiles of the samples and the standards were generated from the images using the visionCATS software (CAMAG, version 2.0) under 366 nm before derivatization. The areas of the peaks of the references and the samples were then used for quantification. The smallest amounts measured were: ferulic acid 0.00029 mg, and Z-ligustilide 0.00566 mg. To ensure comparability of the amounts of ferulic acid and Z-ligustilide of samples between plates, a single-level calibration was conducted for each plate separately.

### 3.4. Statistical Tools and Tests

Statistical analyses were performed using R (R Core Team, 2016, ver. 3.2.5). Unless stated otherwise, the significance level α for all tests was 0.05. The HPTLC analysis showed a lower detection limit of around 0.0003 mg for ferulic acid and 0.01 mg for Z-ligustilide with a number of samples having lower amounts than the detection limit. We thus applied a Tobit regression model using the R package *regr0* with log transformation for the analysis of these two compounds.

### 3.5. Availability of Data and Materials

Raw data are provided in the supplementary information files.

## 4. Results

### 4.1. Cultivation of Angelica Sinensis

In Gansu, the cultivation of *Angelica sinensis* or other medicinal plants is often the main income source, whereas in northwestern Yunnan, it comes from working small jobs and in eastern Yunnan, from cultivation of *Nicotiana tabacum*. Cultivation can be carried out by just one family without special equipment (small-scale household cultivation), particularly in Yunnan, where average field size was 837 m^2^ (SD = 242 m^2^, *n* = 6) in northwestern Yunnan, and 1039 m^2^ (SD: 1118 m^2^, *n* = 5) in eastern Yunnan. In Gansu, fields were significantly larger (*p* = 0.004) with an average of 3996 m^2^ (SD = 2175 m^2^, *n* = 5) and average contribution of *dang gui* to total income of farmers was higher compared to Yunnan (*p* = 0.054; Gansu: 62.5%, SD = 22.2%, *n* = 4; Yunnan: 29.2%, SD = 18.7%, *n* = 5).

In Gansu and northwestern Yunnan, the cultivation of *dang gui* was described by most farmers as a family tradition or a tradition of the whole village. In contrast, in eastern Yunnan, most of the farmers started cultivation less than 15 years ago.

In Gansu, cultivation starts in February or March by planting seedlings from the preceding year. Harvest usually takes place in October. In western Yunnan, the planting and harvest season lasts longer. Interestingly, in eastern Yunnan, all farmers said that they sow the plants in January and harvest in November of the same year.

As essential growing factors, all farmers mentioned altitude (2000–3000 m above sea level) and plenty of rain during seedling growth. Overall, they consider the cultivation as risky due to unpredictable weather and prices. As for most crops, the cultivation of *A. sinensis* is an annual household decision, meaning that farmers trade off their expenses against benefits before cultivation. This in turn means that if the cultivation of *A. sinensis* is no longer considered economic, farmers will switch to other crops instead. In northwestern Yunnan, many famers, even whole villages, gave up the cultivation of *A. sinensis* and started to cultivate other medicinal plants instead.

### 4.2. Trading and Prices

Usually farmers do not process *dang gui* themselves. The whole roots are sold fresh in Gansu and in northwestern Yunnan mainly to local dealers. In eastern Yunnan, they are sold directly to pharmaceutical companies or to the local bureau of agriculture.

Usually, *dang gui* can be sold easily at high prices—much higher than staple foods. However, according to farmers, selling price fluctuates considerably. They think prices are defined by dealers, big companies or by the market in general.

Being asked what they know about the value chain, two farmers in northwestern Yunnan stated that dealers sell *dang gui* in Dali or in Kunming. In Gansu, one farmer said that dealers store *dang gui* to sell it for the best price and to do so they need to smoke it with sulfur. Another farmer in northwestern Yunnan mentioned that middlemen process *dang gui* to get higher profit.

In Kunming, dealers chiefly sell *dang gui* from Gansu. In Shangrila and Lijiang, however, it is mainly the material from Yunnan that is sold. Dealers mentioned that selling prices decreased over the past years, but the demand for *dang gui* has remained unchanged. On average, whole roots are significantly cheaper than heads (M = 222 CNY/kg, SD = 209 CNY/kg, *n* = 21 vs. M = 372 CNY/kg, SD = 255 CNY/kg, *n* = 21; *p* = 0.043). Furthermore, prices are considerably lower in Kunming, compared to the tourist destinations Lijiang and Shangrila.

### 4.3. Quality Notions of Dang Gui

Along the value chain, different criteria were mentioned to be indicative of good quality. For the farmers, size of the root often serves as quality criterion: the bigger the root, the better the quality. Dealers mentioned smell as an important indicator of good quality. For the interviewed doctors, place of cultivation matters most and they regard *dang gui* from Gansu to be the best and more effective in therapy. They argue that the weather in Gansu benefits the quality and that Gansu is the authentic place of production. However, some doctors say that there is no quality difference between *dang gui* from Gansu and Yunnan although smell and taste differ.

### 4.4. Medicinal Use of Dang Gui Along the Value Chain

*Dang gui* is used by all participants of the value chain. Farmers described its medical indications as supplying and quickening the blood and said to frequently use it. In general, they seem to prefer Chinese medicine (CM) over Western medicine (WM) because of less side effects and the ability to “eliminate the root of diseases.”

The dealers told us that customers who buy *dang gui* are mainly women or elderly people who use it to supply blood, move blood or to adjust menstruation.

Approximately half of the lay people (*n* = 14 women, 12 men) have used *dang gui* at least once. It is most often taken as decoction in form of a chicken soup, according to instructions by the doctor.

The CM doctors pointed out that *dang gui* is a frequently used herb for the treatment of anemia, *qi* insufficiency, blood stasis, hematoma and skin ulcer, and that it is most commonly used in combination with other herbs. They emphasized that each part of *dang gui* has a different effect, i.e., the head is used to supplement the blood, the body to supplement and quicken the blood, the tail to quicken the blood, primarily to dispel blood stasis or resolve hematomas. CM doctors most often recommend *dang gui* to be taken as a decoction in form of a soup, but it can also be administered as pill or injection.

### 4.5. Regression of Head Size, Root Length and Provincial Origin with Main Active Compounds

**Figure 3:**
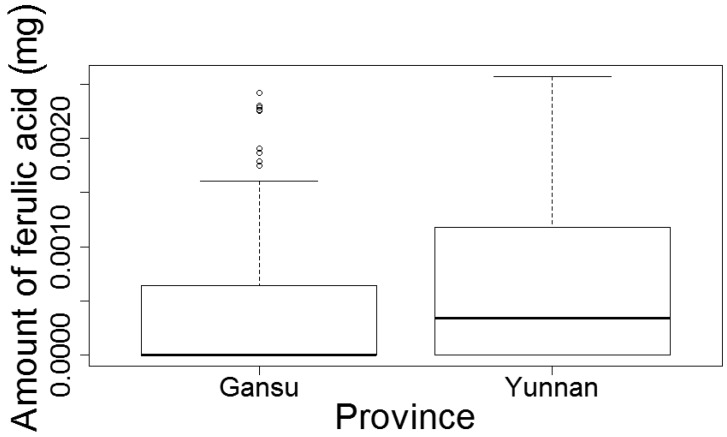
Amounts of ferulic acid of whole roots compared between Gansu and Yunnan Province. Error bars represent the 95% confidential intervals (Gansu, *n* = 111; Yunnan, *n* = 93).

*Dang gui* roots showed a highly significant negative relation between both the size of the head (β; measured as diameter) and the root length (γ) and the amount of ferulic acid (*b* = −0.60, *t* (1) = −1.88, *p* < 0.001; *c* = −0.75, *t* (1) = −4.22, *p* < 0.001). For Z-ligustilide, only root length showed a—also negative—significant relation (*c* = −0.47, *t* (1) = −3.39, *p* < 0.001), while size of the head was not significant (*b* = −0.40, *t* (1) = −0.90, *p* = 0.079). In a regression with “province” as a single explanatory variable, *dang gui* from Yunnan showed significantly higher amounts of ferulic acid than from Gansu province (*t* (1) = −1.11, *p* = 0.024; Figure 3; Data S1). There was no difference in the amount of Z-ligustilide between *dang gui* from Gansu and Yunnan province.

### 4.6. Comparison of Sulfured and Unsulfured Dang Gui

**Figure 4:**
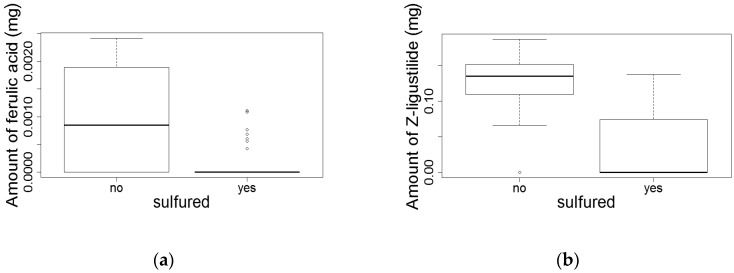
Comparison of (**a**) ferulic acid and (**b**) Z-ligustilide levels between sulfured and unsulfured whole roots. Error bars represent the 95% confidential intervals (sulfured, *n* = 64; unsulfured, *n* = 24). Note: the HPTLC analysis for ferulic acid of sulfured roots yielded a majority of samples below the detection limit.

Ferulic acid and Z-ligustilide are significantly reduced in sulfured roots (Tobit regression for ferulic acid: (*t* (1) = 2.94, *p* < 0.001; for Z-ligustilide: *t* (1) = 2.98, *p* < 0.001; Figure 4; Data S2).

### 4.7. Comparison of Head, Body and Tail

**Figure 5:**
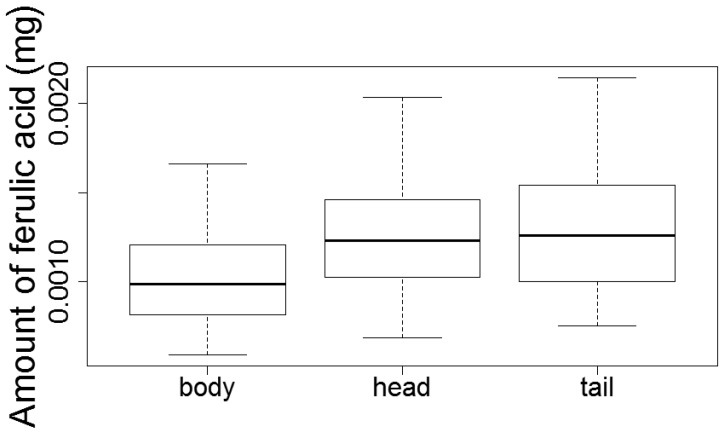
Amounts of ferulic acid between parts of the root. Error bars represent the 95% confidential intervals (head, *n* = 20; body, *n* = 19; tail, *n* = 19).

The quantification shows that there is significant differece of ferulic acid levels within the root with lower amounts found in the body compared to head and tail (*F*(2,55) = 3.45, *p* = 0.04; Figure 5; Data S3). No significant differences were found for Z-ligustilide.

## 5. Discussion

### 5.1. Cultivation and Trading of Dang Gui

Cultivation of *Angelica sinensis* as documented in this study is in large part consistent with recently published Chinese literature [29,30,40]. Its cultivation serves to diversify the income of farmers in quite remote areas of Gansu and northwestern Yunnan where it has a long tradition and hence most farmers are familiar with it. In eastern Yunnan, cultivation is relatively recent. It has been pushed since 2004 by businessmen and by the government, which explains why famers sell their harvest to pharmaceutical companies or the local bureau of agriculture rather than dealers [29].

As high as the profit of *A. sinensis* cultivation can be, its business is just as risky. If the weather is good, the yield increases, but because of a market surplus, the selling price might decrease. This also means that a rich harvest in Gansu may negatively influence selling prices in Yunnan, a pattern also known from other regions in China [41]. In addition, *dang gui* is exported worldwide and is thus linked to foreign markets.

Collaborations between farmers and pharmaceutical companies including secure trade contracts that guarantee fair prices and impose restrictions on the use of pesticides and chemical fertilizers would create incentives to propagate skilled and economic sustainable cultivation [42]. However, it may also limit the decision making of farmers who want to respond flexibly to a fluctuating medicinal plant market.

### 5.2. Quality of Dang Dui and Different Root Parts

Farmers and dealers use size and odor of the root as quality criteria. Dealers sell big roots always at a higher price irrespective of cultivation area. *The American Herbal Pharmacopoeia and Therapeutic Compendium* [25] and the *Materia Medica* [43] also emphasize that good quality consists of thick and long main roots. In Chinese literature, diameter of the main root and odor are mentioned as quality criteria [29]. Z-ligustilide makes up 45%–65% of the essential oil present in *dang gui* and is related to its fragrance [7,25]. Thus, high amounts of Z-ligustilide are probably related with good quality. However, we found no correlation between head diameter and a negative relation of root length and Z-ligustilide as well as a negative relation of both with ferulic acid. The two main marker compounds can thus not explain why a large root size is seen as quality criterion. The emphasis on root size may reflect former wild collection of plant material, when root size correlated with plant age rather than growth conditions of a single vegetation period. This would also explain that one-year old plants sown in January and harvested in December are considered to be of inferior quality [25].

Dealers may process *dang gui* and slice the roots and/or sulfur-smoke them. Both methods have a profound impact especially on the volatile compounds of the drug. We found that ferulic acid and Z-ligustilide were significantly reduced in sulfured roots. Similar results were reported by [6]) using HPLC analysis, and have been described for other crude and prepared drugs entailed by sulfur-smoking [44].

Chinese medicine doctors prefer *dang gui* from Gansu. Interestingly, *dang gui* from Yunnan showed significantly higher amounts of ferulic acid than from Gansu. There was no difference in the amount of Z-ligustilide. However, by means of HPLC, it was shown that *dang gui* from Gansu contained a twofold higher amount of both Z-ligustilide and ferulic acid compared to *dang gui* from Yunnan [6]. Gansu, especially Min County, is traditionally thought to produce the best *dang gui* and considered to be the authentic production region [6,24,40]. It has been shown that differences between cultivation areas are not due to intraspecific genetic diversity, but rather to cultivation and environmental conditions [6,45]. *Angelica sinensis* is a stenotopic species whose natural distribution was mainly confined to places in Gansu and Qinghai [40,46]. Therefore, environmental properties in Gansu might be more suitable for its cultivation compared to Yunnan [47].

According to Chinese medicine and the interviewed doctors, the head, body and tail of *dang gui* are treated as therapeutically distinct parts, as is reflected in the significantly higher price of heads compared to whole roots. Quantification of ferulic acid and Z-ligustilide of head, body and tail showed significant lower ferulic acid levels of the body but no significant differences for Z-ligustilide. Using gas chromatography coupled with mass spectrometry (GC-MS), Wei et al. [2] found significantly higher amounts of ferulic acid and butylidene phthalide in the tail but no differences between head and body.

## 6. Conclusion

This study provides an overview of the complexity of the value chain and helps to develop a more accurate picture of the cultivation and trade of *Angelica sinensis*. Cultivation and trading of *dang gui* is a dynamic business with regions looking back to a long tradition and others having started to cultivate the plant only recently. Prices fluctuate and farmers respond flexibly and adjust the cultivation of different species accordingly. Processing of the drug and storage is carried out by the farmers, or more often by specialized traders or processing facilities, and has a strong influence on the concentration of the marker compounds in the root. Also, different cultivation areas show an influence on the concentration of the marker compounds, but material from Gansu, known as the authentic production area (*Daodi*), had lower concentrations of ferulic acid. Also other quality criteria such as root length and diameter do not correlate with a higher concentration of ferulic acid and Z-ligustilide. This hints at the inherent difficulty to correlate quality notions of medicinal plants with few specific chemical compounds. Moreover, taking into account how common among plants ferulic acid is and that also Z-ligustilide is not exclusive to *dang gui*, their measured amounts are not sufficient for the quality assessment of *dang gui*. With respect to this, more attention should be paid to quality in terms of cultivation and processing, instead of linking quality of herbal medicines only to certain main active compounds. Cultivation practice and processing are fundamental for ensuring both the correct identity and quality of herbal medicines and set the cornerstone for an economic and sustainable value chain.

## Figures and Tables

**Table 1:** Effects and indications of *Angelica sinensis* roots according to Chinese medicine and current pharmacological research.

Use Categories	Chinese Medicine ^1^	Pharmacological Studies	Main Active Compounds
Tonic	Strengthens and invigorates the blood, tonifies the blood		
Gynecological	Regulates the menses; Menstrual disorders like irregular menstruation, amenorrhea and dysmenorrhea		
Cardiovascular	Activates blood circulation; Blood deficiency with symptoms such as dizziness and palpitations	Improves blood fluidity and inhibits platelet aggregation [11,12,13] Cardiovascular and cerebrovascular diseases [12,14]	Ferulic acid, Z-Ligustilide, *A. sinensis* extract Z-Ligustilide
Respiratory	Disperses cold; Chronic bronchitis		
Gastrointestinal	Moistens (unblock) the intestines and relaxes the bowels; Constipation, abdominal pain		
Nervous system	Alleviates pain		
Other	Rheumatism; Sores, abscesses	Anticancer activities [15,16] Antioxidant activities [14,17,18] Anti-inflammatory effects [17,18,19] Potential treatment of diabetes mellitus [1,20] Alzheimer’s disease [18,21,22,23]	Polysaccharides Z-Lugustilide, *A. sinensis* extract, Polysaccharides Polysaccharides, *A. sinensis* extract Ferulic acid, Z-Ligustilide Polysaccharides, ferulic acid

^1^ According to Chinese pharmacopoeia [3,24,25].

**Table 2:** Interviews in Gansu and Yunnan Province.

Province	Location	Prefecture Level	Inter-Views
Gansu	Huichuan Town (Huìchuānzhèn)	Dingxi City (Dìngxī Shì)	4
	Fuzishan Village (Fǔzǐshāncūn)	Dingxi (Dìngxī)	1
Yunnan	Machang (Mǎchǎng)	Dali Bai Autonomous Prefecture (Dàlǐ Báizú Zìzhìzhōu)	5
	Lameirong (Lāměiróng)	Lijiang City (Lìjiāng Shì)	1
	Labadi (Làbādǐ)	Diqing Tibetan Autonomous Prefecture (Díqìng Zàngzú Zìzhìzhōu)	3
	Tacheng (Tǎchéng)	Lijiang City (Lìjiāng Shì)	1
	Bole (Bōlè)	Qujing City (Qǔjìng Shì)	2
	Yanfang (Yánfāng)	Qujing City (Qǔjìng Shì)	2
	Lingjiao (Língjiǎo)	Qujing City (Qǔjìng Shì)	2
	Baishui (Báishuǐ)	Qujing City (Qǔjìng Shì)	1
	Kunming (Kūnmíng)	Kunming City (Kūnmíng Shì)	96

## Supplementary Materials

The following are available online at www.mdpi.com/2305-6320/4/1/14/s1, Data S1: Regression, Data S2: Sulfured, Data S3: Head, body, tale.
